# Supplementary material for: How Does the Context Shape the Technical Support from the Provincial Health Administration to District Health Management Teams in the Democratic Republic of Congo? A Realist Evaluation
Source: Int J Environ Res Public Health. 2024 Dec 10;21(12):1646. doi: 10.3390/ijerph21121646 (PMC11675160; doi:10.3390/ijerph21121646)
Supplement: Supplementary file 1 [file ijerph-21-01646-s001.zip › S1. National performance assessment of PHAs in 2021.pdf]

Supplemental File S1. Performance assessment of provincial health administrations in 2021.

Extracted from the annual report of the health sector 2021 (and translated into English).

Comparative analysis of the performance of the provincial health administrations (PHAs) in 2021 in relation to the monitoring indicators of the National Health Development Plan (NHDP) 2019-2022

Out of the 40 indicators in the Monitoring and Evaluation Framework for the NHDP 2019-2022, only 20 are monitored annually. The table below displays the performance of the PHAs based on 12 indicators for which data were entered into the DHIS2.

Table S1. Performance scores by indicator and by PHA in 2021.

NAME OF THE ORGANISATION UNIT	Rate of use of services (outpatient consultations)	Proportion of references	Intra-hospital mortality rate beyond 48 hours	Proportion of maternal deaths in hospital audited	Proportion of ANC 4 at 36 weeks	BCG vaccination coverage rate	VAR vaccination coverage rate	Pentavalent 3 vaccination coverage rate	Overall completeness of DHIS2 reports	Overall accuracy of NHIS reports	DHIS2 data quality score	PHA performance in 2021 at 30 November 2021
**Bas Uele**	2.5	0	3.5	4	3	1.5	2.5	2.5	4	1.5	2.5	63%
**Equateur**	4	2	3	4	3.5	2	3	3	2.5	1.5	2	69%
**Haut Katanga**	2.5	0	3	4	3	3.5	4	4	3	1.5	2	69%
**Haut Lomami**	3	2	3.5	4	4	3.5	3.5	4	2.5	1.5	1.5	75%
**Haut Uele**	3	2	3	4	3	1.5	2.5	3	3	1.5	2	65%
**Ituri**	3	2	3.5	4	3.5	2	3.5	4	3	1.5	2	73%
**Kongo Central**	3.5	2	3	4	2.5	1.5	3.5	3.5	3.5	3	3	75%
**Kasai Oriental**	3	2.5	3	4	4	1.5	3.5	3.5	4	2.5	3.5	80%
**Kwango**	4	2	3.5	4	4	3	4	4	2.5	2	2	80%
**Kwilu**	3.5	2	3.5	4	4	2	3.5	3.5	4	3	3.5	83%
**Kinshasa**	2.5	0	2	0	2.5	3	3	3	3.5	2.5	3	57%
**Kasai Central**	4	4	3.5	4	4	3.5	4	4	3.5	2.5	3	91%
**Kasai**	4	0	3.5	4	4	2	4	4	3.5	2.5	2.5	77%
**Lualaba**	4	0	3	4	3.5	2.5	3.5	3.5	2	1.5	1.5	66%
**Lomami**	2.5	4	3	4	4	2.5	3.5	3.5	4	3	3.5	85%
**Maindombe**	3.5	2	3.5	4	4	2.5	3	3	2.5	1	1	68%
**Mongala**	4	2.5	3.5	4	4	2	3.5	3.5	2.5	1.5	2	75%
**Maniema**	3	2	3.5	4	4	2	3	3	3	1.5	2	70%
**Nord Kivu**	4	4	3.5	4	4	1.5	3.5	3.5	2.5	1.5	2	77%
**Nord Ubangi**	4	4	3	4	4	2	3	3	2.5	1.5	1	73%
**Sud Kivu**	4	4	3.5	4	3.5	2	3.5	3.5	3	1.5	2	78%
**Sankuru**	3	4	3.5	4	4	1.5	3.5	3.5	2.5	1.5	2	75%
**Sud Ubangi**	4	2	3	4	4	1	2.5	2.5	4	2	3	73%
**Tanganyika**	1.5	0	3	4	3.5	2	2.5	2.5	2.5	1	1	53%
**Tshopo**	2.5	0	3.5	4	3	1.5	2	2	2.5	1	1.5	53%
**Tshuapa**	4	2	3	4	4	3	3.5	3	3.5	3	3	82%
**RDC 2021**	3.5	2	3	4	3.5	2.5	3.5	3.5	3	2	2.5	75%
DRC Performance indicators in 2021	83%	49%	81%	96%	91%	54%	82%	83%	76%	47%	56%	

Comments:

In 2021, eleven indicators were benchmarked. At the country level, the analysis revealed that two indicators performed very well: The proportion of maternal deaths in hospitals audited/accounting for maternal deaths (96%) and the Proportion of ANC 4 to 36 weeks (91%). Four indicators performed well: Service utilization rate (83%), In-hospital mortality rate beyond 48 hours (81%), VAR 2021 vaccination coverage rate (82%), and Pentavalent 3 2021 vaccination coverage rate (83%). One indicator was average: Overall completeness of reports in DHIS2 (76%); Two indicators were weak: BCG Vaccine Coverage Rate 2021 (54%) and DHIS2 data quality score (56%); Two indicators were very weak: proportion of references (49%) and overall timeliness of national health information system (NHIS) reports (47%).

It should also be noted that the country's performance in 2021 (75%) improved compared with previous years. It increased compared with 2020 (63%), 2018 (63%), and 2019 (66%).

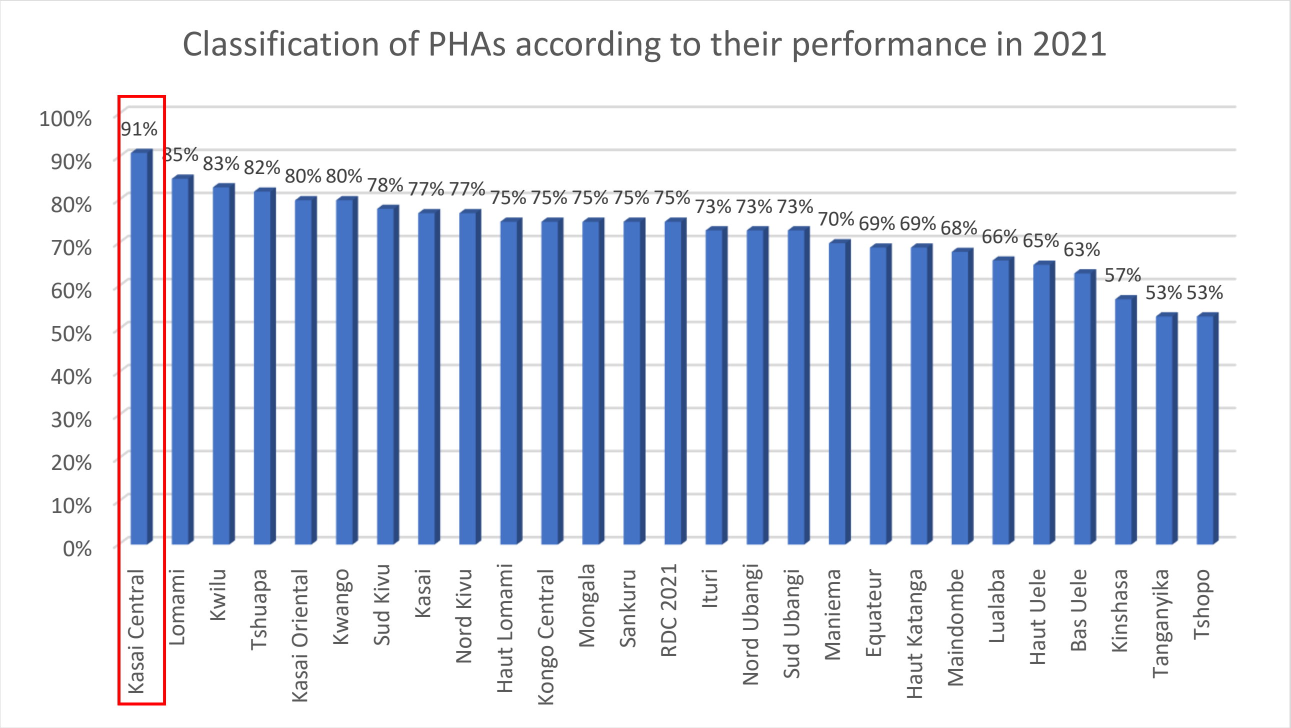

Figure S1. Classification of the performance of PHAs according to NHDP indicators in 2021
