# Supplementary material for: How Does the Context Shape the Technical Support from the Provincial Health Administration to District Health Management Teams in the Democratic Republic of Congo? A Realist Evaluation
Source: Int J Environ Res Public Health. 2024 Dec 10;21(12):1646. doi: 10.3390/ijerph21121646 (PMC11675160; doi:10.3390/ijerph21121646)
Supplement: Supplementary file 1 [file ijerph-21-01646-s001.zip › S2. Scorecard_DHMT management capacity.pdf]

Supplementary File S2. Scorecard for assessing the management capacities of DHMTs.

1- Instructions

1. Explain to the DHMT members the research's objectives and data collection process
2. Request to the head of health district for DHMT member to work with (preferably the DHMT secretary).
3. Request the following documents for the year 2022, archiving permitting:
    - Internal rules of the DHMT
    - Designation of DHMT members
    - Distribution of responsibilities within the DHMT
    - Job-description of each DHMT member
    - Minutes of weekly DHMT meetings
    - Minutes of monthly Health District Management Committee meetings
    - Minutes of Health District Administration Board
    - Consolidated operational action plan for the health district
    - Operational action plans for DHMT, district hospital and health centres
    - Quarterly work plans for DHMT
    - Reports of health district annual and half-yearly review
    - Monthly activity reports of health district office
    - Training needs of health district workforce
    - Training plan for health district workforce
    - Reports on training sessions organised in the health district
    - Reports of post-training follow-up sessions
    - Terms of reference for training, supervision, monitoring and other activities
    - Reports on supervisions carried out by the DHMT members
    - Stock sheets for routine data collection tools
    - Copies of monthly feedback to health facilities
    - Minutes of monthly data analysis meetings
    - Minutes of weekly epidemiological surveillance meetings
    - Epidemic investigation reports
    - Health district all-hazards contingency plan
    - Declarative list of health district workforce
    - Annual staff appraisal reports
    - Health district management resource procedures manual
    - Financial management tools (bank and cash books, cash receipts and disbursements, financial repor
    - List of health district materials and equipment
    - Materials and equipment maintenance plan
    - List of essential generic drugs
    - Drug management tools
    - Drug monthly inventory reports
    - Research reports

4. Screen these documents to assign scores to each managerial function according to the criteria in the scorecard below.

5. To assign a score, the DHMT must meet all the criteria corresponding to that score; if not, the next lower score is assigned.

6. Throughout the completion process, discuss success and failure factors, focusing on technical support's role, and note comments.
7. For each function, the DHMT's management capacity is calculated as a percentage ratio between the obtained score and the maximum score.

   The overall DHMT's management capacity is calculated as a percentage ratio between the total obtained score and the maximum score (112).
8. Classify the DHMT's management capacity as follows:
   - Good: 80 -100%
   - Average: 50-79,9%
   - Low: < 50%
9. Thank the DHMT member(s) for their availability and cooperation.

2- Scorecard

Health district [ ] **Province** [ ] **Year** [ ]

**Management functions**	**Instructions**	**Description of scoring criteria**				**Assigned score**
		**Score 1**	**Score 2**	**Score 3**	**Score 4**	
**1. Coordination** (12 points)						
**Organisation and operation of the health district management team (DHMT)**	*Check the existence of internal rules, letters of appointment, the allocation of responsibilities and job descriptions for DHMT members.*	DHMT has no internal rules.	DHMT has no internal rules.	DHMT has internal rules drawn up in a participatory manner and available at the health district (HD) office.	DHMT has internal rules drawn up in a participatory manner, approved by the head of PHA and available at the HD office.	
		DHMT members are not formally appointed by the head of PHA.	DHMT members are not formally appointed by the head of PHA.	DHMT members are formally appointed by the head of PHA.	DHMT members are formally appointed by the head of PHA.	
		All DHMT members have vertical or horizontal responsibilities officially assigned to them by the hierarchy.	All DHMT members have vertical or horizontal responsibilities officially assigned to them by the hierarchy.	All DHMT members have vertical or horizontal responsibilities officially assigned to them by the hierarchy.	DHMT have a consensual distribution of vertical and horizontal responsibilities among for each member.	
		All DHMT members do not have job descriptions archived or posted at the HD office.	Some DHMT members have job descriptions archived or posted at the HD office.	All DHMT members have job descriptions archived or posted at the HD office.	All DHMT members have job descriptions archived or posted at the HA office.	
	Check HD office's monthly reports for the number of DHMT meetings held with archived minutes (standard: 52 meetings/year).	Less than 60% of DHMT meetings were held with archived minutes at the HD office.	Between 60-79% of DHMT meetings were held with archived minutes at the HD office.	Between 80-99% of DHMT meetings were held with archived minutes at the HD office.	100% of DHMT meetings were held with archived minutes at the HD office.	

**Functionality of the management committee (MACO) of health district**	*Check HD office's monthly reports for the number of MACO meetings held with archived minutes (standard: 12 meetings/year).*<br>*Check the implementation of decisions/ recommendations.*	Less than 6 MACO meetings were held with or without archived minutes at the HD office.	Between 6-11 MACO meetings were held with archived minutes at the HD office.	12 MACO meetings were held with archived minutes at the HD office.<br>Less than 100% of decisions/ recommendations from MACO meetings are implemented.	12 MACO meetings were held with archived minutes at the HD office.<br>100% of decisions/ recommendations from MACO meetings are implemented.	
**Functionality of administration broad (AB) of health district**	*Check HD office's monthly reports for the number of AB meetings held with archived minutes (standard: 12 meetings/year).*<br>*Check the implementation of decisions/ recommendations.*	No AB meeting was held with archived minutes at the HD office.	One AB meetings was held with archived minutes at the HD office.	Two AB meetings were held with archived minutes at the HD office.<br>Less than 100% of decisions/ recommendations from AB meetings were implemented.	Two AB meetings were held with archived minutes at the HD office.<br>100% of decisions/ recommendations from AB meetings were implemented.	
**2. Planning, Monitoring et Evaluation** (12 points)						
**Development of operational action plans (AOP)**	*Check that the HD's is available and was developed in a bottom-up manner (by consolidating the OAPs from DHMT, district hospital and health centres) and in a participatory manner (by involving the various stakeholders).*	The OAP is not available at the HD office.	The OAP is available in electronic form and not available to all DHMT members.	The OAP is available in hard copy, archived in the HD office and available to all DHMT members.	The OAP is available in hard copy, archived in the HD office and available to all DHMT members.<br>The OAP was developed in a bottom-up and participatory manner: AOPs from DHMT, district hospital and health centres and attendance lists of planning workshop participants are archived at the HD office.	

	Check that the HD's AB approved the OAP.				The OAP was approved by the HD's AB with archived minutes.	
**Monitoring of OAP implementation**	*Check that the OAP was broken down into quarterly work plans (QWPs) for its implementation.*<br>*Check that the HD's MACO approved the QWPs.*	The OAP is not at all declined in QWP: no QWP archived at the HD office.	The OAP is not systematically declined in QWP: less than 4 QWP archived at the HD office	The OAP is systematically declined in QWP: 4 QWP archived at the HD office	The OAP is systematically declined in QWP: 4 QWP archived at the HD office<br>All QWPs were approved by the HD's MACO with archived minutes.	
**Evaluation of the OAP**	*Check that the AOP was evaluated during the HD's biannual and annual reviews (standard: 2 reviews/year) or at any other opportunity.*<br>*Check that mid-term and annual OAP evaluations were approved by the HD's AB..*	No reviews (biannual and annual) have been organised to evaluate the OAP: no evaluation report archived at the HD office.	A review (biannual or annual) has been organised to evaluate the OAP, with a report archived at the HD office.	Two reviews (biannual or annual) have been organised to evaluate the OAP, with reports archived at the HD office.	A review (biannual or annual) has been organised to evaluate the OAP, with a report archived at the HD office.<br>Two AB meetings were held to approve the OAP mid-term and annual evaluations, with the minutes archived at the HD office.	
**3. Hands-on training** (12 points)						
**Identification of training needs**	*Ask DHMT members who identifies training needs and how?*<br>*Check that the HD has a documented training plan.*	Training needs of HD's workforce are not at all known or documented.	Training needs are identified or dictated by the PHA office or funders.	Training needs are identified by the DHMT without a skills assessment.	Training needs are identified by the DHMT on the basis of a skills assessment.<br>The DHMT has a training plan on file at the HD office.	

**Scheduling and execution of training**	*Ask DHMT members who plans training. Check the level of completion of scheduled training in the HD's monthly reports*	Training sessions were scheduled and run unilaterally by the PHA office and/or funders.	Scheduling and execution of training sessions were negotiated with the PHA office and funders.	Less than 80% of training sessions scheduled by the DHMT were completed.	More than 80% of training sessions scheduled by the DHMT were completed.	
**Post-training follow-up**	*Check HD office's monthly reports for the number of training and post-training follow-up organised, with reports archived.*	No follow-up was organised on any training sessions: no reports were filed.	Less than 50% post-training follow-up were organised, with reports archived at the HD office.	More than 50% post-training follow-up were organised, with reports archived at the HD office.	100% of post-training follow-up were organised, with reports archived at the HD office.	
**4. Supportive supervision** (12 points)						
**Planning of supervision**	*Ask DHMT members how supervision topics are identified.*<br><br>*Check that supervision plans and TORs were discussed and validated at DHMT meetings (standard: 1 meeting/month)*	DHMT planned supervision based on funders' priority, and not on deficiencies identified through analysis of health information or previous supervision.	DHMT planned supervision based on identified deficiencies from health information or previous supervision.<br><br>Not all supervision plans have been discussed and validated within the DHMT: no minutes attest to this.	DHMT planned supervision based on identified deficiencies from health information or previous supervision.<br><br>Some supervision plans have been discussed and validated within the DHMT: less than 12 minutes attest to this.	DHMT planned supervision based on identified deficiencies from health information or previous supervision.<br><br>All supervision plans have been discussed and validated within the DHMT: 12 minutes attest to this.	
**Implementation and quality of supervision**	*Check HD's monthly reports for the number of supervision visits carried out with archived reports (standard: 1 supervision/facility/ month).*	DHMT carried out less than 60% of planned supervision visits with reports archived at the HD office.	DHMT carried out 60-79% of planned supervision visits with reports archived at the HD office.	DHMT carried out 80-99% of planned supervision visits with reports archived at the HD office.	DHMT carried out 100% of planned supervision visits with reports archived at the HD office.	

	Analyse a few supervision reports to assess their quality.			Poor quality supervision: discrepancies between weaknesses and recommendations, vague, imprecise and irrelevant recommendations, poor quality of reporting.	Good quality supervision: concordance between weaknesses and recommendations, relevant, specific and precise recommendations, good reporting.	
**Evaluation of supervision**	*Inquire whether supervision visits are evaluated each month at the DHMT meetings, and check few minutes to ensure that lessons learned are documented and considered in future visits (standard: 1 meeting/month).*	DHMT did not evaluated any supervision visit: no minutes attest to this.	DHMT evaluated some supervision visits: less than 12 minutes attest to this.	DHMT evaluated some supervision visits: less than 12 minutes attest to this.	DHMT evaluated some supervision visits: less than 12 minutes attest to this.<br><br>Lessons learned are drawn from each evaluation and considered in future supervision.	
**5. Health information management** (12 points)						
**Availability of data collection tools**	*Inquire whether health facilities in the HD experienced stock-outs of data collection tool, and estimate the approximate number of facilities that have experienced stock-outs during the year.*	More than half the health facilities in the HD experienced stock-outs of data collection tools (forms, registers, reporting templates) during the year.	Half the health facilities in the HD experienced stock-outs of data collection tools (forms, registers, reporting templates) during the year.	A quarter of the health facilities in the HD experienced stock-outs of data collection tools (forms, registers, reporting templates) during the year.	No health facilities in the HD experienced stock-outs of data collection tools (forms, registers, reporting templates) during the year.	
**Data analysis and feedback**	*Check HD's monthly reports for the number of data analysis meetings, (standard: 12 meetings/year).*<br><br>*Check that written feedback were sent to health facilities (standard: 1 feedback/facilities/ month).*	Less than 60% of data analysis meetings were held, with minutes archived.	Between 60-79% of data analysis meetings were held, with minutes archived.	Between 80-99% of data analysis meetings were held, with minutes archived.<br><br>DHMT sent at least 60% of written feedback to health facilities, with copies archived at the HD office.	100% of data analysis meetings were held, with minutes archived.<br><br>DHMT sent at least 80% of written feedback to health facilities, with copies archived at the HD office.	

**Use of health information**	*Request the ToRs of various activities, including planning, training, supervision and check the reference to health information*	DHMT did not use health information at all in the planning, training, supervision and decision-making processes: no reference to health information in the ToRs checked.	DHMT sometimes used health information in the planning, training, supervision and decision-making processes: reference to health information in less than 50% of the ToRs checked.	DHMT often used health information in the planning, training, supervision and decision-making processes: reference to health information in more than 50% of the ToRs checked.	DHMT systematically used health information in the planning, training, supervision and decision-making processes: reference to health information in all ToRs checked.	
**6. Epidemiological surveillance** (12 points)						
**Analysis of epidemiological surveillance data**	*Check HD's monthly reports for the number of epidemiological surveillance meetings, (standard: 52 meetings/year).*	Less than 60% of epidemiological surveillance meetings were held, with minutes archived at the HD office.	Between 60-79% of epidemiological surveillance meetings were held, with minutes archived at the HD office.	Between 80-99% of epidemiological surveillance meetings were held, with minutes archived at the HD office.	100% of epidemiological surveillance meetings were held, with minutes archived at the HD office.	
**Investigation of suspected cases of diseases and events under surveillance**	*Check HD's monthly reports for the proportion of suspicious cases of diseases and events under surveillance investigated and documented (standard: 52 meetings/year).*	Less than 60% of notified suspicious cases of diseases and events under surveillance were investigated and documented.	Between 60-79% of notified suspicious cases of diseases and events under surveillance were investigated and documented.	Between 80-99% of notified suspicious cases of diseases and events under surveillance were investigated and documented.	100% of notified suspicious cases of diseases and events under surveillance were investigated and documented.	
**Contingent epidemics**	*Check that the HD's contingency plan is available and was developed in a participative manner, involving various stakeholders*	The HD does not have an all-hazards contingency plan.	The HD has an all-hazards contingency plan in electronic form, not available to all DHMT members.	The HD has an all-hazards contingency plan on paper, available to all DHMT members.<br><br>The contingency plan was not developed using a participatory process: there were no attendance lists for the contingency plan workshop.	The HD has an all-hazards contingency plan on paper, available to all DHMT members.<br><br>The contingency plan was developed using a participatory process: there were attendance lists for the contingency plan workshop.	
**7. Human resource management** (8 points)						

**Mastering the number and needs of HD's workforce**	*Check that the HD has a workforce database.*<br><br>*Check that personnel files are filed.*<br><br>*Check that HD's staffing needs are known and documented.*	DHMT does not have an up-to-date database (electronic or paper) of HD workforce.	DHMT has an out-of-date database (electronic or paper) of HD workforce.	DHMT has an up-to-date database (electronic or paper) of HD workforce.<br><br>The files of some HD health workers are filed at the HD office.	DHMT has an up-to-date database (electronic or paper) of HD workforce.<br><br>The files of some HD health workers are filed at the HD office.<br><br>The HD's staffing needs are known, documented and used as advocacy tools with the PHA.	
**HD staff appraisal**	*Check whether DHMT has carried out annual appraisal of HD staff, with archived reports.*	DHMT has not carried out any annual appraisals of HD staff on the basis of the criteria laid down in the legal administrative texts.	DHMT carried out annual appraisals for less than half of HD staff, based on the criteria laid down in legal administrative texts.	DHMT carried out annual appraisals for more than half of HD staff, based on the criteria laid down in legal administrative texts.	DHMT carried out annual appraisals of all HD staff on the basis of the criteria laid down in the legal administrative texts.	
**8. Financial resource management** (8 points)						
**Budget management**	*Check that the DHMT had an annual budget that included all sources of funding.*	DHMT did not have an annual budget that included all sources of funding.	DHMT had an annual budget that included all sources of funding, but did not the budget monitoring.	DHMT had an annual budget that included all sources of funding for the ZS, did the budge monitoring but did not systematically document it.	DHMT had an annual budget that included all sources of funding for the ZS, did the budge monitoring and systematically documented it.	
**Financial management procedures**	*Check that the DHMT had a financial management procedures manual and that these procedures were respected, including financial management tools.*	DHMT did not have a financial management procedures manual.	DHMT had the financial management procedures manual but did not comply with it.	DHMT had the financial management procedures manual and complied with it.<br><br>Financial management tools existed but were not up to date.	DHMT had the financial management procedures manual and complied with it.<br><br>Financial management tools existed and were up to date.	
**9. Material resource management** (8 points)						

**Mastering of needs and inventory of materials, equipment and infrastructure**	*Check that DHMT had a database of HD's materials, equipment and infrastructure and that inventories are carried out (standard: 1 inventory/year).*	DHMT did not have a database of the HD's materials, equipment and infrastructure.	DHMT had a partial database of the HD's materials, equipment and infrastructure.	DHMT had a complete database of the HD's materials, equipment and infrastructure.<br>The HD's material, equipment and infrastructure needs were known and documented	DHMT had a complete database of the HD's materials, equipment and infrastructure.<br>The HD's material, equipment and infrastructure needs were known and documented.<br>DHMT carried out an annual inventory of the HD's materials, equipment and infrastructure for all facilities.	
**Maintenance of materials, equipment and infrastructure**	*Check that DHMT carried out maintenance of the HD's materials, equipment and infrastructure.*	DHMT did not performed any maintenance of the HD's materials, equipment or infrastructure: no archived reports.	DHMT sporadically performs maintenance of some HD's materials, equipment and infrastructure, with no archived reports.	DHMT sporadically performs maintenance of some the HD's materials, equipment and infrastructure, with archived reports.	DHMT performed and documented the maintenance of the HD's materials, equipment and infrastructure on the basis of a maintenance plan.	
**10. Drug management** (12 points)						
**Selection and quantification essential drug needs**	*Check that DHMT had a local list of essential medicines and mastered the HD's drug requirements.*	DHMT did not have a local list of essential drugs drawn up on the basis of the epidemiological profile of the HD, and did not master the needs for essential drugs in the HD.	DHMT did not have a local list of essential drugs drawn up on the basis of the epidemiological profile of the HD, but had an estimate of the HD's needs for certain drugs, particularly those for vertical programmes.	DHMT had a local list of essential drugs drawn up on the basis of the epidemiological profile of the HD, but did not master the HD's needs for essential drugs, except for those of certain vertical programs.	DHMT had a local list of essential drugs drawn up on the basis of the epidemiological profile of the HD and master the HD's needs for essential drugs.	
**Procurement of essential drugs**	*Ask DHMT members how medicines were procured in the HD. Check the objectivity of their answers by examining supply*	DHMT did not supply any health facilities with essential drugs: each facility supplied itself in its own way.	DHMT supplied some health facilities with essential drugs from certain funders and vertical programmes.	DHMT supplies all health facilities with essential drugs from all funders and vertical programmes.	DHMT supplies all health facilities with essential drugs from all funders and vertical programmes.	

	documents: invoices, delivery notes, receipt slips.			DHMT supplied certain health facilities with essential drugs through group purchases from the regional distribution centre.	DHMT supplied all health facilities with essential drugs through group purchases from the regional distribution centre.	
**Stock management of essential drugs**	*Check that the DHMT performed monthly drug inventories at the HD office and updated stock sheets.*	DHMT performed and documented less than 6 monthly drug inventories at the HD office.	DHMT performed between 6-8 monthly drug inventories at the HD office, with archived reports.	DHMT performed between 9-11 monthly drug inventories at the HD office, with archived reports.<br>Some of the items available at the HD office's pharmaceutical depot had up-to-date stock sheets.	DHMT performed 12 monthly drug inventories at the HD office, with archived reports.<br>Some of the items available at the HD office's pharmaceutical depot had up-to-date stock sheets.	
**11. Research** (4 points)						
	Check that DHMT conducted and documented operational or action research aimed at improving the quality of health care and services.	DHMT did not identified and documented any operational or action research related to the quality of care and services or any other health issue in the HD.	DHMT identified and documented a few operational or action research topics related to the quality of care and services or other health issues in the HD.	DHMT developed one or more operational or action research protocols related to the problems identified.	DHMT conducted at least one operational or action research project to improve the quality of health care and services in the HD.	

Inspired from Kahindo JB, Meessen B, Byamungu T et al. Contrat de performance avec une administration sanitaire provinciale: cas du Nord Kivu en République Démocratique du Congo. PBF CoP Working Paper Series. WP3. 2011

3- **Results**

**Management functions**	KATOKA		BUNKONDE	
	**Score**	**%**	**Score**	**%**
**1. Coordination** (12 points)	**8**	**67%**	**7**	**58%**
Organisation and operation of the health district management team	2	50%	2	50%
Functionality of the management committee of health district	3	75%	2	50%
Functionality of administration broad of health district	3	75%	3	75%
**2. Planning, Monitoring et Evaluation** (12 points)	**11**	**92%**	**11**	**92%**
Development of operational action plans (OAP)	3	75%	3	75%
Monitoring of OAP implementation	4	100%	4	100%
Evaluation of the OAP	4	100%	4	100%
**3. Hands-on training** (12 points)	**7**	**58%**	**3**	**25%**
Identification of training needs	4	100%	1	25%
Scheduling and execution of training	2	50%	1	25%
Post-training follow-up	1	25%	1	25%
**4. Supportive supervision** (12 points)	**8**	**67%**	**8**	**67%**
Planning of supervision	4	100%	4	100%
Implementation and quality of supervision	3	75%	3	75%
Evaluation of supervision	1	25%	1	25%
**5. Health information management** (12 points)	**12**	**100%**	**11**	**92%**
Availability of data collection tools	4	100%	4	100%
Data analysis and feedback	4	100%	3	75%
Use of health information	4	100%	4	100%
**6. Epidemiological surveillance** (12 points)	**8**	**67%**	**6**	**50%**
Analysis of epidemiological surveillance data	3	75%	4	100%
Investigation of suspected cases of diseases and events under surveillance	4	100%	1	25%
Contingency if epidemics	1	25%	1	25%

**7. Human resource management** (8 points)	**4**	**50%**	**4**	**50%**
Mastering the number and needs of health district's workforce	3	75%	3	75%
Health district staff appraisal	1	25%	1	25%
**8. Financial resource management** (8 points)	**5**	**63%**	**8**	**100%**
Budget management	4	100%	4	100%
Financial management procedures	1	25%	4	100%
**9. Material resource management** (8 points)	**4**	**50%**	**5**	**63%**
Mastering of needs and inventory of materials, equipment and infrastructure	2	50%	2	50%
Maintenance of materials, equipment and infrastructure	2	50%	3	75%
**10. Drug management** (12 points)	**10**	**83%**	**10**	**83%**
Selection and quantification essential drug needs	2	50%	2	50%
Procurement of essential drugs	4	100%	4	100%
Stock management of essential drugs	4	100%	4	100%
**11. Research** (4 points)	**1**	**25%**	**1**	**25%**

**Total**	**78**	**70%**	**74**	**66%**
