# Supplementary material for: How Does the Context Shape the Technical Support from the Provincial Health Administration to District Health Management Teams in the Democratic Republic of Congo? A Realist Evaluation
Source: Int J Environ Res Public Health. 2024 Dec 10;21(12):1646. doi: 10.3390/ijerph21121646 (PMC11675160; doi:10.3390/ijerph21121646)
Supplement: Supplementary file 1 [file ijerph-21-01646-s001.zip › S3. Interview guides.pdf]

Supplementary File S3. Interview guides.

2.1. Interview guide for provincial health administration staff

Elements	Actions / Questions / Remarks
**Introduction**	Arrival and installation<br>Explain the purpose of the interview<br>Explain the process of the interview (see information sheet)<br>Guarantee anonymity and confidentiality<br>Ask for the interview to be recorded and notes to be taken.<br>Obtain the informed consent<br>Install and check the recording material
**Beginning of the interview**	First, I will ask you a few general questions to get an idea of your profile.
**Profile of the participant**	Q: How old are you?<br>Q: What is your professional qualification (doctor, nurse, midwife, etc.)?<br>Q: Could you please provide a summary of your career progression, including your seniority level and the positions you have held?<br>Q: How long have you been in your current position?<br>Q: What are your main responsibilities?
**Transition**	Let's talk about your understanding and the process of technical support to the DHMTs.
**Understanding and practice of technical support**	Q: What does technical support for the DHMT consist of?<br>Q: Can you explain how the technical support visits to the DHMTs are conducted in reality? (Probe: explore the activities/tasks carried out before, during and after these visits)
**Need-driven technical support**	Q: In your opinion, why should support be focused on the needs of the DHMTs members?<br>Q: In practice, how do you identify these needs? (Probe: explore the participatory aspect of the process)<br>Q: Do vertical programs and funders have an impact on this process? If yes, how do you ensure that the support provided stays relevant to the needs of the DHMT members?
**Problem-solving approach**	Q : Selon vous, pourquoi l'appui technique devrait adopter une approche de résolution des problèmes auxquels sont confrontés les membres des ECZS ?<br>Q : Comment procédez-vous en pratique pour apporter un appui à la résolution des problèmes aux membres des ECZS ?<br>Q : Dans quelle mesure votre approche de résolution des problèmes contribue-t-elle au renforcement des capacités des membres des ECZS ?
**Reflexions, feedback and learning**	Q: Why do you think it's important for the provincial coach to support the DHMTs in learning through constructive reflection and feedback on their management practices?<br>Q: In practice, how do you stimulate these reflections? Can you give a concrete example?<br>Q: What are the factors that influence learning within the DHMT? (Probe: explore receptiveness to feedback, open discussions without fear of judgement or blame, the relationship of trust, etc.).

**Regularity of technical support**	Q: How often are technical support visits to the health district made to your PHA?<br>Q: And how long does a visit last, on average?<br>Q: Can you list the factors that affect (both positively and negatively) the frequency and duration of technical support visits to the health zones, both positively and negatively? (Probing: explore the availability of supervisors, financial and logistical issues, and scheduling conflicts).
**Transition**	Let's talk now about your competencies, motivation and relationships with the DHMTs.
**Competences of provincial coach**	Q: Why is the gradient of competence between the provincial coach and the DHMT members important?<br>Q: Do you think you have a higher gradient of competences compared to the DHMT members you supervise?<br>Q: How do you work on improving your competences? (Probe: training, meetings)<br>Q: How do you ensure that DHMT members receive support on topics where you may not have the required expertise?
**Motivation of provincial coach**	Q: What motivates you to work as a provincial coach? (Probe: explore the elements of intrinsic and extrinsic motivation)<br>Q: Have you considered leaving the public service? If so, why? If not, why not?<br>Q: Do you think you are providing additional work beyond your responsibilities?<br>If so, why do you do it? (Probe: financial remuneration or other incentives, compliance with hierarchical orders, conscientiousness) Can you give examples?<br>If not, why?
**Relationship with DHMTs**	Q: Can you share your thoughts on what makes a good relationship between the provincial supervisor and the DHMT members in the context of technical support? And why is this relationship significant?<br>Q: To what extent does your hierarchical position (PHA staff) affect (positively or negatively) your relationship with the DHMT members you supervise?<br>Q: What do you do in the event of conflicts with DHMT members?
**Transition**	Let us talk now about the outcomes of technical support to the DHMTs.
**Outcomes of technical support**	Q: Do you think that technical support, as currently conducted, is contributing to achieving results (i.e., strengthening DHMT members' skills and improving health districts' management and performance)?<br>If so, can you give concrete examples in terms of:<br>- Individual skills of the DHMT members?<br>- Organisation and functioning of the DHMTs?<br>- Health district management practices?<br>- Health district performance?<br>Q: If not, why not?
**Announcement of the end**	We are almost at the end of our interview, but I have some final questions before we finish...
**Miscellaneous**	During this interview, you provided valuable information regarding the process and expected outcomes of technical support from provincial coaches to DHMTs.<br>Q: If you were asked to formulate recommendations for improving technical support to health districts, what would they be?<br>Q: Is there anything else regarding technical support that you would like to share with me? If there is, we have enough time to discuss it right now.<br>Q: Do you have any questions you would like to ask me?
**End of the interview**	Thank you for being available. I would like to assure you that your answers are confidential. If I need more information, I will contact you without hesitation.

2.2. Interview guide for DHMT members

Elements	Actions / Questions / Remarks
**Introduction**	Arrival and installation<br>Explain the purpose of the interview<br>Explain the process of the interview (see information sheet)<br>Guarantee anonymity and confidentiality<br>Ask for the interview to be recorded and notes to be taken.<br>Obtain the informed consent<br>Install and check the recording material
**Beginning of the interview**	First, I will ask you a few general questions to get an idea of your profile.
**Profile of the participant**	Q: How old are you?<br>Q: What is your professional qualification (doctor, nurse, midwife, etc.)?<br>Q: Could you please provide a summary of your career progression, including your seniority level and the positions you have held?<br>Q: How long have you been in your current position?<br>Q: What are your main responsibilities?
**Transition**	Let's talk about your understanding and the process of technical support to the DHMTs.
**Understanding and practice of technical support**	Q: What does technical support for the DHMT consist of?<br>Q: What do the provincial coaches actually do during their technical support visits to your health district? (Probe: explore the activities/tasks carried out before, during and after these visits)
**Need-driven technical support**	Q: Why do you think technical support should be focused on your needs?<br>Q: In concretely, how does your provincial coach identify these needs? (Probing: explore the participative aspect of the process)<br>Q: Are you involved in identifying these needs?<br>Q: How does your involvement or lack of involvement in identifying your support needs influence your attitude toward the technical support process?<br>Q: Can you tell me about the influence of vertical programmes and funders in identifying your support needs?
**Problem-solving support**	Q: In your opinion, why should technical support adopt a problem-solving approach to your problems?<br>Q: In practice, how does your coach support you in solving problems?<br>Q: To what extent does your coach's problem-solving approach help to build your capacity?<br>Q: How does this approach influence your attitude to the technical support process?
**Reflexions, feedback and learning**	Q: In your opinion, why should the provincial coach facilitate learning within the DHMTs through constructive reflection and feedback on management practices?<br>Q: In practice, how does your provincial coach stimulate these reflections? Can you give a concrete example?<br>Q: Do you feel comfortable expressing your views without fear of being judged or blamed by others (coach, head of health district or colleagues)?<br>Q: To what extent do these reflections contribute to strengthening your skills and improving the performance of your health district?<br>Q: How do you appreciate the feedback from your provincial coach?

**Regularity of technical support**	Q: How often are technical support visits made to your health district?<br>Q: How long do these visits last, on average?<br>Q: Are these visits regular? If not, why?
**Transition**	Let us talk now about your perception of the competencies and relationships with your provincial coach.
**Competences of provincial coach**	Q: How do you appreciate the competence gradient of your provincial coach?<br>Q: To what extent does this appreciation influence your attitude to the technical support process?
**Relationship with DHMTs**	Q: Can you share your thoughts on what makes a good relationship between the provincial supervisor and the DHMT members in the context of technical support? And why is this relationship significant?<br>Q: How do you appreciate your relationship with your provincial coach?<br>Q: How does this relationship influence your attitude in the technical support process?<br>Q: How do you manage conflicts with your provincial coach?
**Transition**	Let us talk now about your motivation and the outcomes of technical support you received from your provincial coach.
**Motivation of DHMT members**	Q: What motivates you to work as a DHMT member? (Probing: explore elements of intrinsic and extrinsic motivation)<br>Q: Have you considered leaving the public service? If yes, why? If not, why not?<br>Q: Do you feel you are providing additional work beyond your responsibilities?<br>If yes, why do you do it? (Probing: financial remuneration or other incentives, respect for hierarchical orders, professional conscience) Can you give examples?<br>If not, why not?<br>Q: How do you appreciate the contribution of technical support to your motivation?
**Individual competences of DHMT members**	Q: Overall, how would you rate your own management and/or leadership skills?<br>Q: Do you think you are capable of effectively performing certain managerial functions (e.g. planning, supervision, health information management, etc.) independently?<br>Q: How do you assess the contribution of technical support in strengthening your individual skills?
**DHMT management practices**	Q: How do you rate the organisation and operation of your DHMT? (Probing: explore the distribution of responsibilities, job description, regular meetings, information flow, collegial decision-making, cohesion and mutual support, etc.).<br>- How do you assess the contribution of technical support to the organisation and operation of your DHMT?<br>- How do the organisation and operation of your DHMT influence your attitude (satisfaction, motivation, commitment) at work?<br>Q: How do you assess the general management of your health district? (Probing: explore aspects of planning, monitoring & evaluation, supervision, ongoing training, resource management, etc.).<br>- How do you assess technical support's contribution to managing your health district?<br>- What other factors influence management practices in your health district, and how? (Probing: explore the autonomy required for management, availability of resources, political interference)<br>- How does the management of your health district influence your attitude (satisfaction, motivation, commitment) at work?

**Health district performances**	Q: Overall, how do you rate the performance of your health district?<br>Q: How do you appreciate the contribution of technical support in improving the performance of your health districts?<br>Q: What other factors influence the performance of your health district, and how?
**Announcement of the end**	We are almost at the end of our interview, but I have one or two final questions before we finish...
**Miscellaneous**	During this interview, you provided valuable information regarding the process and expected outcomes of technical support from provincial coaches to DHMTs.<br>Q: If you were asked to formulate recommendations for improving technical support to health districts, what would they be?<br>Q: Is there anything else regarding technical support that you would like to share with me? If there is, we have enough time to discuss it right now.<br>Q: Do you have any questions you would like to ask me?
**End of the interview**	Thank you for being available. I would like to assure you that your answers are confidential. If I need more information, I will contact you without hesitation.
