# Supplementary material for: How Does the Context Shape the Technical Support from the Provincial Health Administration to District Health Management Teams in the Democratic Republic of Congo? A Realist Evaluation
Source: Int J Environ Res Public Health. 2024 Dec 10;21(12):1646. doi: 10.3390/ijerph21121646 (PMC11675160; doi:10.3390/ijerph21121646)
Supplement: Supplementary file 1 [file ijerph-21-01646-s001.zip › S4. Questionnaire .pdf]

Supplementary File S4. Questionnaire for district health management team (DHMT) members.

Dear respondent,

Thank you for agreeing to complete this questionnaire. It should take at most 15-20 minutes to complete. Before you begin, we want to reassure you that:

1) Your answers will remain confidential and only accessible to the research team.
2) There are no right or wrong answers. We value your opinion and appreciate your honesty.

1. Socio-demographic characteristics of DHMT members

1.1	Name of health district *(write down ✍ the name)*	___________________________
1.2	Sex *(tick ☑ the appropriate response)*	☐ Male ☐ Female
1.3	How old are you? *(write down ✍ your age)*	______ years
1.4	What level of education do you have? *(tick ☑ the appropriate response)*	☐ Secondary<br>☐ Graduate (BAC+3)<br>☐ Licence/Master (BAC+5 or more)
1.5	What is your current position? *(tick ☑ the appropriate response)*	☐ Head of health district<br>☐ Head of district hospital<br>☐ Chief of medical staff<br>☐ Administrator<br>☐ Nurse Supervisor<br>☐ Director of Nursing<br>☐ Pharmacist<br>☐ Nutritionist<br>☐ Sanitation technician<br>☐ Other (please specify): _____________________
1.6	How long have you worked in the healthcare system? *(write down ✍ the duration)*	______ years
1.7	How long have you been in your current position? *(write down ✍ the duration)*	______ years
1.8	Did you work in a district health management team before your current position? *(tick ☑ the appropriate response)*	☐ Yes<br>☐ No
1.9	Have you been trained in primary healthcare management or any other related management and/or leadership training? *(tick ☑ the appropriate response)*	☐ Yes<br>☐ No (*go to part 2*)
1.10	If yes, in which year? *(write down ✍ the training year)*	

2. Perceptions of DHMT members on the technical support from the Provincial Health Administration (PHA)

For each statement below, please express your opinion by ticking ☑ the box corresponding to your level of agreement or disagreement.

		Strongly disagree	Disagree	Neutral	Agree	Strongly agree
2.1	Our provincial coaches have the required skill set to provide us with effective technical support.	☐	☐	☐	☐	☐
2.2	Technical support visits to our health district are regular and of sufficient duration.	☐	☐	☐	☐	☐
2.3	Technical support visits to our health district are carried out according to a consensual support plan agreed upon in advance.	☐	☐	☐	☐	☐
2.4	Our provincial coaches share relevant information on the DHMT members through standards, guidelines and other national and provincial documents.	☐	☐	☐	☐	☐
2.5	Technical support visits are based more on our support needs and the problems we face.	☐	☐	☐	☐	☐
2.6	The technical support visits are more dictated by the priorities of the specialised programmes funded by the external donors.	☐	☐	☐	☐	☐
2.7	Our provincial coaches help us to solve the problems we face during the technical support visits.	☐	☐	☐	☐	☐
2.8	Our provincial coaches challenge us to reflect on our practices/performance and encourage us to freely express our ideas or opinions without being judged or blamed.	☐	☐	☐	☐	☐
2.9	Our provincial coaches provide us with relevant, constructive and practical feedback to improve our individual and collective performance.	☐	☐	☐	☐	☐
2.10	Our provincial coaches respect us, listen to us, recognise our efforts, support us and motivate us to achieve our mission.	☐	☐	☐	☐	☐
2.11	The support from our provincial coaches contributes to strengthening individual managerial capacities.	☐	☐	☐	☐	☐
2.12	The support from our provincial coaches helps to improve the organisation, functioning, cohesion and performance of our DHMT.	☐	☐	☐	☐	☐
2.13	The technical support of our provincial coaches helps to improve the performance of our health district.	☐	☐	☐	☐	☐
2.15	Overall, the technical support provided in our health district is useful and is bringing about positive changes.	☐	☐	☐	☐	☐

2.14	What are your expectations of technical support at provincial level? *(write down ✍ your expectation)*	

Many thanks for your time and contribution!
