# Supplementary material for: How Does the Context Shape the Technical Support from the Provincial Health Administration to District Health Management Teams in the Democratic Republic of Congo? A Realist Evaluation
Source: Int J Environ Res Public Health. 2024 Dec 10;21(12):1646. doi: 10.3390/ijerph21121646 (PMC11675160; doi:10.3390/ijerph21121646)
Supplement: Supplementary file 1 [file ijerph-21-01646-s001.zip › S5. Scorecard_HD performance.pdf]

Supplementary File S5. Scorecard and calculation of the performance of health districts using benchmarking method.

Step 1. Selection of key performance indicators (KPIs)

We selected ten KPIs from the monitoring and evaluation framework of the National Health Development Plan (NHDP) 2019-2020. These KPIs served as a proxy for measuring access, quality and equity of health care and services. These indicators and their operational definitions are outlined below:

#	Domains	Indicators	Numerator	Denominator
1	Reporting	Overall completeness rate	Total number of reports received from health facilities	Total number of reports expected from health facilities
2	Access	Curative service use rate	Number of people who consulted curative care services	Total number of the population
3	Access	Antenatal care 4 coverage (ANC4)	Number of pregnant women who have completed four antenatal care visits	Total number of the expected pregnant women (4% of population)
4	Access	Skilled birth attendance (SBA)	Number of pregnant women who have gave birth in the presence of a skilled personnel	Total number of the expected pregnant women (4% of population)
5	Access	Contraceptive prevalence	Number of women of childbearing age who received a modern contraceptive method	Total number of women of childbearing age (21% of the population)
6	Access	DTC-HepB-Hib3 coverage	Number of children under 12 months who received 3 doses of the DTC-HepB-Hib vaccine	Total number of surviving children under 12 months (3,49% of population)
7	Quality	In-hospital mortality rate > 48 hours	Number in-hospital death beyond two hospitalisation day	Total number of number of inpatients admitted
8	Quality	Tuberculosis treatment success rate	Number of people who completed their tuberculosis treatment	Total number of tuberculosis patients enrolled in treatment cohort
9	Equity	Percentage of HIV+ pregnant women put on ART	Number of VIH+ pregnant women put on antiretroviral treatment	Number of VIH+ pregnant women diagnosed
10	Equity	Percentage of children under five with malaria treated according to the national protocol	Number of under-5 children with confirmed malaria treated according to the national guideline	Total number of under-5 children with confirmed malaria

Step 2. Assigning targets to KPIs

For each KPI selected, we assigned a target from the monitoring and evaluation framework of the national development plan 2019-2020, as follows:

#	Key Performance Indicators	NHDP Target 2022
1	Overall completeness rate	100%
2	Curative service use rate	50%
3	Antenatal care 4 coverage (ANC4)	61%
4	Skilled birth attendance (SBA)	88%
5	Contraceptive prevalence	13%
6	DTC-HepB-Hib3 coverage	96%
7	In-hospital mortality rate > 48 hours	4%
8	Tuberculosis treatment success rate	94%
9	Percentage of HIV+ pregnant women put on ART	76%
10	Percentage of children under five with malaria treated according to the national guidelines	100%

Source: National Health Development Plan 2019-2022

Step 3. Developing the scorecard

For each KPI, we assigned a score from 0 (very poor) to 4 (very good) based on its target. This scoring was divided into 9 standards, as shown in the table below.

#	Key Performance Indicators	NHDP Target 2022	Scores									
			**0**	**0,5**	**1**	**1,5**	**2**	**2,5**	**3**	**3,5**	**4**	ascending order
			**4**	**3,5**	**3**	**2,5**	**2**	**1,5**	**1**	**0,5**	**0**	descending order
1	Overall completeness rate	**100%**	0%	13%	25%	38%	50%	63%	75%	88%	100%	
2	Curative service use rate	**50%**	0%	6%	13%	19%	25%	31%	38%	44%	50%	
3	Antenatal care 4 coverage (ANC4)	**61%**	0%	8%	15%	23%	31%	38%	46%	53%	61%	
4	Skilled birth attendance (SBA)	**88%**	0%	11%	22%	33%	44%	55%	66%	77%	88%	
5	Contraceptive prevalence	**13%**	0%	2%	3%	5%	7%	8%	10%	11%	13%	
6	DTC-HepB-Hib3 coverage	**96%**	0%	12%	24%	36%	48%	60%	72%	84%	96%	
7	In-hospital mortality rate > 48 hours	**4%**	0,0%	0,5%	1,0%	1,5%	2,0%	2,5%	3,0%	3,5%	4,0%	
8	Tuberculosis treatment success rate	**94%**	0%	12%	24%	35%	47%	59%	71%	82%	94%	
9	Percentage of HIV+ pregnant women put on ART	**76%**	0%	10%	19%	29%	38%	48%	57%	67%	76%	
10	Percentage of children under five with malaria treated according to the national guidelines	**100%**	0%	13%	25%	38%	50%	63%	75%	88%	100%	

How to score?

For the curative service use rate, for example, we proceeded as follows to assign scores:

- Score 0 : if the actual indicator is less than 6%
- Score 0,5 : if the actual indicator is ≥6% and <13%
- Score 1 : if the actual indicator is ≥23% and <19%
- Score 1,5 : if the actual indicator is ≥19% and <25%
- Score 2 : if the actual indicator is ≥25% and <31%
- Score 2,5 : if the actual indicator is ≥31% and <38%
- Score 3 : if the actual indicator is ≥38% and <44%
- Score 3,5 : if the actual indicator is ≥44% and <50%
- Score 4 : if the actual indicator is ≥50%

Step 4. Data collection and KPI calculation

We collected data from the DHIS2 software and calculated the actual KPIs according to their operational definitions outlined in Step 1.

Health district 1. KATOKA

#	Key Performance Indicators	NHDP Target 2022	Actuals				
			2018	2019	2020	2021	2022
1	Overall completeness rate	**100%**	30,6%	35,7%	49,9%	67,9%	98,9%
2	Curative service use rate	**50%**	69%	65%	69%	64%	56%
3	Antenatal care 4 coverage (ANC4)	**61%**	95%	94%	93%	85%	70%
4	Skilled birth attendance (SBA)	**88%**	97%	99%	100%	97%	89%
5	Contraceptive prevalence	**13%**	32%	30%	32%	29%	27%
6	DTC-HepB-Hib3 coverage	**96%**	90%	101%	98%	98%	98%
7	In-hospital mortality rate > 48 hours	**4%**	1,3%	1,2%	1,3%	0,7%	2,3%
8	Tuberculosis treatment success rate	**94%**	89%	96%	92%	92%	97%
9	Percentage of HIV+ pregnant women put on ART	**76%**		18%	92%	21%	100%
10	Percentage of children under five with malaria treated according to the national protocol	**100%**	97,0%	99,0%	99,3%	99,9%	99,9%

Source: DHIS2, accessed on 21-12-2023

Health district 2. Bunkonde

#	Key Performance Indicators		Actuals

		NHDP Target 2022	2018	2019	2020	2021	2022
1	Overall completeness rate	**100%**	29,4%	36,1%	42,4%	81,1%	99,4%
1	Curative service use rate	**50%**	59%	53%	53%	52%	52%
2	Antenatal care 4 coverage (ANC4)	**61%**	90%	86%	87%	85%	80%
3	Skilled birth attendance (SBA)	**88%**	107%	87%	77%	76%	74%
4	Contraceptive prevalence	**13%**	5%	6%	7%	11%	7%
5	DTC-HepB-Hib3 coverage	**96%**	95%	95%	99%	98%	95%
6	In-hospital mortality rate > 48 hours	**4%**	1,0%	1,0%	1,0%	1,0%	0,0%
7	Tuberculosis treatment success rate	**94%**	96,8%	94,6%	94,4%	94,1%	94,7%
9	Percentage of HIV+ pregnant women put on ART	**76%**	0,0%	0,0%	0,0%	0,0%	100,0%
10	Percentage of children under five with malaria treated according to the national protocol	**80%**	91,5%	89,0%	91,9%	86,2%	96,2%

Source: DHIS2, accessed on 21-12-2023

Step 5. Scoring and calculation of the performance of the health districts

We used the scorecard developed in Step 2 to assign each KPI a score based on its actual level. For each year, we calculated the synthetic performance indicator as a percentage ratio between the total score obtained by the health district and the maximum score expected (40). We classified the performance of health districts as follows:

- Good: 80 -100%
- Average: 50-79,9%
- Low: 50%

Health district 1. KATOKA

#	Key Performance Indicators	NHDP Target 2022	Scores				
			**2018**	**2019**	**2020**	**2021**	**2022**
1	Overall completeness rate	**100%**	1	1	1,5	2,5	3,5
2	Curative service use rate	**50%**	4	4	4	4	4
3	Antenatal care 4 coverage (ANC4)	**61%**	4	4	4	4	4
4	Skilled birth attendance (SBA)	**88%**	4	4	4	4	4
5	Contraceptive prevalence	**13%**	4	4	4	4	4
6	DTC-HepB-Hib3 coverage	**96%**	3,5	4	4	4	4
7	In-hospital mortality rate > 48 hours	**4%**	3	3	3	3,5	2
8	Tuberculosis treatment success rate	**94%**	3,5	4	3,5	3,5	4
9	Percentage of HIV+ pregnant women put on ART	**76%**	0	0,5	4	1	4
10	Percentage of children under five with malaria treated according to the national protocol	**100%**	3,5	3,5	3,5	3,5	3,5
	Total		**29,5**	**31**	**34**	**31,5**	**33,5**
	Percentage		**74%**	**78%**	**85%**	**79%**	**84%**

Health district 2. BUNKONDE

#	Key Performance Indicators	NHDP Target 2022	Scores				
			2018	2019	2020	2021	2022
1	Overall completeness rate	**100%**	1	1	1,5	3	3,5
1	Curative service use rate	**50%**	4	4	4	4	4
2	Antenatal care 4 coverage (ANC4)	**61%**	4	4	4	4	4
3	Skilled birth attendance (SBA)	**88%**	4	3,5	3,5	3	3
4	Contraceptive prevalence	**13%**	1,5	1,5	2	3	2
5	DTC-HepB-Hib3 coverage	**96%**	3,5	3,5	4	4	3,5
6	In-hospital mortality rate > 48 hours	**4%**	3	3	3	3	4
7	Tuberculosis treatment success rate	**94%**	4	4	4	4	4
9	Percentage of HIV+ pregnant women put on ART	**76%**	0	0	0	0	4
10	Percentage of children under five with malaria treated according to the national protocol	**80%**	3,5	3,5	3,5	3	3,5
	Total		**27,5**	**27**	**28**	**28**	**32**
	Percentage		**69%**	**68%**	**70%**	**70%**	**80%**
